# Analysis of health-related, skill-related physical strength, and physique by blood pressure levels of Korean seniors

**DOI:** 10.1371/journal.pone.0279264

**Published:** 2022-12-30

**Authors:** Aram Kim, Eunjung Kim, Seunghui Baek, Jieun Shin, Seungwook Choi

**Affiliations:** 1 Department of Sport Leisure, Sungshin Women’s University, Seoul, Korea; 2 Department of Health Exercise Management, Sungshin Women’s University, Seoul, Korea; 3 Department of Biomedical Informatics, College of Medicine, Konyang University, Seoul, Korea; Universiti Malaya, MALAYSIA

## Abstract

In Korea, the prevalence rate of hypertension among people aged over 30 is on an upward trend, which increased from 26.2% in 2008 to 28.3% in 2018. This hovers above the global morbidity rate of hypertension. As hypertension is the cause of cerebrovascular or cardiovascular diseases, early treatment and management are critical. Also, while there has been numerous research conducted on exercise intervention, the number of studies done on hypertension and physical fitness falls far below. To identify the relationship between health and physical fitness depending on the blood pressure levels of Korean seniors, the physical fitness test results of the National Fitness Award 100 conducted in 2017 were used, and blood pressure level, physique, and physical fitness data of 17,110 elderly population aged above 65 were analyzed. IBM Statistics SPSS 25.0 was used to process the collected data. Due to the gender difference in blood pressure levels, physique, and physical strength, the analysis was conducted by each gender, and the analysis of variance (ANOVA) was performed to identify the differences in physique and physical strength depending on the blood pressure level. Also, Tukey’s HDS test was conducted if such differences were found. All analyzes were tested at the significance level (α) of 0.05. As a result of comparing the physique factors by blood pressure level, only males showed a significant difference between groups in terms of height(p = .019), and higher blood pressure implied greater height. Also, for both genders, those with high blood pressure appeared to have greater body weight(p < .001), body fat percentage(p < .001), BMI(p < .001), waist circumference(p < .001), and waist-to-height ratio(p < .001),. In addition, as the result of comparing health-related physical strength and skill-related physical strength by blood pressure level, males showed a significant difference only in muscular strength(p = .026) and flexibility(p < .001), while females showed a significant difference between groups in terms of cardiovascular endurance(p < .001), muscular strength(p = .025), muscular endurance(p < .001), balance, and motor coordination except for flexibility. Holistically, males only showed a significant difference in muscular strength and flexibility while females appeared to have a significant difference between groups in all categories except flexibility, which can be considered that physical fitness factors influence the blood pressure of females more than males.

## 1 Introduction

The improvement and changes in lifestyle with the growing economy have also transformed the lifestyle and eating habits of people. These changes have led to medical problems linked to metabolic syndromes such as high blood pressure, dyslipidemia, hyperglycemia and obesity [1].

Hypertension, in particular, is one of the major causes of death along with diseases related to our brain, heart, kidney, and others [2]; as hypertension can lead to myocardial infarction, stroke, and kidney failure if left untreated in the early stage [3–5], its prevention has been highlighted as a key public health issue across the world [6]. The situation has been particularly dire in Asia, where the prevalence of hypertension has risen rapidly in comparison with heart disease and stroke [7]. For example, in Korea, the prevalence rate of hypertension among the population aged 30 or above increased by 2.7%p from 24.5% in 2007 to 27.7% in 2019 [8], which hovers above the global hypertension morbidity. Also, hypertension is one of the most common diseases among Korean seniors as can be seen by the prevalence rate reaching 46.0% among those aged 60 or above and 70.2% among those aged 70 or above as of 2018.

Genetic characteristics, gender, age, smoking, drinking, overweight, and lack of physical activity [9] were found to be the causes of hypertension, and in Korea, male, the older, low income level, smoking, drinking, high body mass index, neutral fat were suggested as risk factors for hypertension electricity [10]. For adults over 25 years of age, improving their lifestyle and habits can lower blood pressure by 5~10mmHg [11], but unfortunately, most people try to control their high blood pressure with drugs [12].

The Korean Society of Hypertension and American Heart Association provide a guideline that healthy lifestyle must be combined with taking medication to effectively manage hypertension [13, 14]. In managing hypertension, factors that can correct some of the causes of hypertension include practicing health behaviors such as abstinence from alcohol and cigarette, regular exercise, and weight management [14–17]. To manage hypertension, it is important to receive medical treatment as well as reducing risk factors of brain cardiovascular disease by correcting living habits [18–22], and the practice of these health behaviors is expected not only to reduce complications caused by hypertension but also to improve the quality of life of the elderly with hypertension [23, 24].

Physical activity, one of the ways to improve hypertension, not only lowers blood pressure directly [25] but also helps improve body weight and body fat [26]. It is also important as it contributes to the enhancement of health-related physical strength such as cardiovascular endurance, muscular strength, muscular endurance, and flexibility [27].

Also, as an overall integrated measure of physical function(skeletal muscle, cardiopulmonary, blood circulation, neuropsychology, and endocrine metabolism) related to daily physical activity or exercise performance, physical fitness is a predictor of causes and mortality of cardiovascular disease [28–31] that hypertension is one of the major determinants of cardiovascular morbidity and mortality as a decrease in SBP 10mmHg in the elderly reduces mortality by 13%, chronic heart disease deaths by 18%, and stroke by 26% [32–34].

As physical activity and physical strength are interlinked like a chain, more engagement in physical activity leads to better physical strength. In other words, regular aerobic exercise helps improve cardiovascular endurance while lowering blood pressure [35–38], and can also prevent most metabolic diseases including cardiovascular diseases and type 2 diabetes [39–41]. However, on the other hand, as the loss of physical strength can lead to less physical activity, it is critical for hypertensive patients to establish such a virtuous cycle. For seniors, in particular, it is necessary to make efforts to boost physical strength to reduce the risk of developing various diseases and extend life expectancy [42, 43] as good physical strength is relevant to chronic diseases and mortality rates [44].

While many international and national organizations have been recommending seniors to carry out the physical activity at least for a minimal amount to maintain health, let alone numerous studies about exercise intervention conducted to identify the importance of physical activity and preventing hypertension, there have not been enough in-depth studies on blood pressure and physical strength.

Therefore, based on the data from the “National Fitness Award 100” of the Korea Sports Promotion Foundation (KSPO), which has been leading the measurement of physical strength by life cycle, this study aimed to identify physical strength factors that can be helpful for the improvement of senior blood pressure and look at the relationship between Korean seniors’ blood pressure level and physical strength. It also aimed to provide primary data for the establishment of exercise programs for seniors by blood pressure, accustomed to different situations depending on necessary physical strength factors.

## 2 Materials and methods

### 2.1. Data and subjects

The study utilized the physical fitness test results of the “2017 National Fitness Award 100” provided by the Korea Sports Promotion Foundation (KSPO). The National Fitness Award of KSPO is an official certification authority designated by the government, which officially performs physical fitness measurement, physical fitness evaluation, exercise prescription, and physical fitness certification. The subjects were seniors aged 65 or above. The total number of subjects was 17,110 (11,855 females, 5,255 males), and the average age was 71.8 for females and 72.7 for males. This experimental protocol was approved by the Institutional Review Committee of Sungshin Women’s University (SSWUIRB-2021-036) and informed to and received the consent of all participants. The characteristics of the subjects are as follows.

### 2.2. Blood pressure standard

The blood pressure levels were divided into four groups based on the standards of the Korean Society of Hypertension: below 120/88mmHg as Normal, 120-129/80mmHg as Elevated, 130-139mmHg systolic pressure or 80-89mmHg diastolic pressure as Prehypertension, and 140mmHg or above systolic pressure or 90mmHg or above diastolic pressure as Hypertension [45].

### 2.3. Physique and obesity index

For physique, an automatic weight/height scale (BSM370, In-Body, Korea) was used to measure the height (cm), body weight (kg), and body mass index (kg/m^2^). Waist circumference (cm) was measured with a tape measure, and body fat percentage was measured with a body composition analyzer (InBody 620, In-Body, Korea) [46].

### 2.4. Physical fitness

Health-related physical strength (cardiorespiratory endurance, muscular strength, muscular endurance, flexibility) and skill-related physical strength (balance, motor coordination) were measured for physical fitness [46].

#### 2.4.1 Cardiorespiratory endurance (two-minute walk-in-place)

The minimum height to which the subject has to raise their knees was set for each subject. Then, the distance between the middle of the knee to the ilium (iliac crest) was measured with a tape measure, after which a tape was used to mark the middle of the femur. With the starting signal, the subject began with their right leg, tagging one knee after another to the rubber string. It was measured as “one time” when both of the legs fully walked for two minutes starting with their right foot. A chance was given for the subject to practice before the measurement, and there were no second measures.

#### 2.4.2 Muscular strength (grip strength)

The subject was ordered to hold a hand gripper with second knuckles of their hand, stretch out their arm forward keeping their body and arm distanced by 15 degrees and grip it as hardest as they could for five seconds with the posture unchanged. The left and right arms were measured twice one after another, and the best score of each was recorded by 0.1kg unit. Then, relative handgrip strength (handgrip strength ÷ body weight ×100) was calculated.

#### 2.4.3 Muscular endurance (sit-to-stand)

The subject was instructed to sit in the center of the chair with their back straight and both arms crossed at the wrists and gathered in front of the chest. Then on the mark, the subject was told to fully stand up, and return to a fully seated position. They were encouraged to repeat it as many as they could in 30 seconds, and the total count of full sit-and-stands performed for 30 seconds was recorded. If the subject was in a half-stood position when 30 seconds were over, it was counted as a full stand-up. The measurement was only allowed once.

#### 2.4.4 Flexibility (sit-and-reach)

The subject was instructed to take off their shoes and sit with knees stretched forward until the soles were placed flat against the measurement device. Then, the subject was told to keep their knees pressed flat to the floor with palms touching the box facing downwards, and bend the upper body and extend it as far forward as possible. Measurement was performed twice, and the better record was selected and recorded in units of 0.1cm.

#### 2.4.5 Balance (timed up and go)

A cone was installed at exactly 3m-point from the back of the cone to the frontal edge of the chair (one spot on the floor). The chair was placed against the wall so that it faces the cone, and the subject was instructed to stand up, walk around the cone, then return to the chair to the seated position as fast as they could with the starting signal. The subject was ordered to repeat it twice and was given only one chance for the measurement. The fastest time was recorded in units of 0.1 seconds.

#### 2.4.6 Motor coordination (figure-of-8 walk)

A 3.9m (width) × 1.6m (length) rectangle was drawn, and cones were fixed within the side edges, where a chair was placed at the 2.4m-point from them. The subject was told to stand by, sitting on the chair at the center of the frontal edge of the rectangle, after which was instructed to stand, go around the cone on the right, return to the seated position, stand up right after again, go around the cone of the left, and return to the seated position, with the “start” signal. This course was repeated twice, and the record was measured in units of 0.1 seconds.

### 2.5 Statistical analysis

IBM Statistics SPSS 25.0 was used to process the collected data. Due to the gender difference in blood pressure levels, physique, and physical strength, the analysis was conducted by each gender, and the analysis of variance (ANOVA) was performed to identify the differences in physique and physical strength depending on the blood pressure level. Also, Tukey’s HDS test was conducted if such differences were found. All analyzes were tested at the significance level (α) of 0.05.

## 3 Results

### 3.1 Analysis of blood pressure by gender

In terms of blood pressure, 41.6% of female subjects were classified into the Normal group, 37.2% in the Elevated group, 16.9% in the Prehypertension group, and 4.4% in the Hypertension group.

For males, 38.5% were classified into the Normal group, 36.4% in the Elevated group, 19.4% in the Prehypertension group, and 5.7% in the Hypertension group. Males showed slightly higher figures in the Prehypertension and Hypertension groups compared to their female counterparts (Table 1).

**Table 1:** Distribution and age of each blood pressure group by gender.

	Female (n = 11,855)	Male (n = 5,255)
**Normal**	**(41.6)71.3±5.2**	**(38.5)72.7±5.4**
**Elevated**	**(37.2)72.2±5.3**	**(36.4)73.0±5.4**
**Prehypertension**	**(16.9)72.0±5.5**	**(19.4)72.1±5.3**
**Hypertension**	**(4.4)72.1±5.4**	**(5.7)72.6±5.7**

(%), Mean±SD

### 3.2 Analysis of differences in physique and body fat percentage

The study looked at the differences in height, body weight, BMI, waist circumference, waist-to-height ratio, and body fat percentage by blood pressure level. It was found that male seniors showed a statistically significant difference in all physique factors, while female seniors also showed a statistically significant difference in all factors except height.

In terms of males, for body height, the Hypertension group (165.95±6.00) was the tallest, while the Normal group (164.97±5.77) was the shortest(p = .019). Meanwhile, body weight increased with higher blood pressure(p < .001). In short, males with higher blood pressure had higher height with greater body weight. For obesity rate measured with waist circumference and waist-to-height ratio, the Elevated group, Prehypertension group, and Hypertension group showed a high figure. For waist circumference, it was 85.35±7.84 for the Elevated group, 86.34±7.59 for the Prehypertension group, and 86.77±9.76 for the Hypertension group. Meanwhile, for the waist-to-height ratio, the Elevated group recorded 0.54±0.05, the Prehypertension group 0.55±0.05, and the Hypertension group 0.55±0.06 (Table 2). For body fat percentage, the Prehypertension group and Hypertension group recorded 26.46±6.06 and 26.94±6.11, respectively. For BMI, it was 24.61±2.7 for the Prehypertension group and 24.95±2.63 for the Hypertension group. In short, the two groups, Prehypertension and Hypertension, showed high figures in body fat percentage(p < .001) and BMI(p < .001), measures of obesity.

**Table 2:** Analysis of differences in physique by male blood pressure group.

		N	Mean	*SD*	*F*	*p*
**Height (cm)**	**Normal**	**2023**	**164.97** ^**a**^	**5.77**	**3.331**	**.019**
	**Elevated**	**1911**	**165.24** ^**ab**^	**6.03**		
	**Prehypertension**	**1019**	**165.47** ^**ab**^	**5.99**		
	**Hypertension**	**300**	**165.95** ^**b**^	**6.00**		
	**Total**	**5253**	**165.22**	**5.93**		
**Body Weight (kg)**	**Normal**	**2024**	**64.24** ^**a**^	**8.52**	**47.650**	**< .001**
	**Elevated**	**1910**	**65.98** ^**b**^	**8.48**		
	**Prehypertension**	**1019**	**67.43** ^**c**^	**8.53**		
	**Hypertension**	**300**	**68.79** ^**d**^	**8.65**		
	**Total**	**5253**	**65.75**	**8.63**		
**BMI (kg/m** ^**2**^ **)**	**Normal**	**2024**	**23.59** ^**a**^	**2.81**	**45.945**	**< .001**
	**Elevated**	**1908**	**24.13** ^**b**^	**2.61**		
	**Prehypertension**	**1019**	**24.61** ^**c**^	**2.70**		
	**Hypertension**	**300**	**24.95** ^**c**^	**2.63**		
	**Total**	**5251**	**24.06**	**2.74**		
**Waist Circumference (cm)**	**Normal**	**1113**	**83.31** ^**a**^	**8.88**	**23.903**	**< .001**
	**Elevated**	**1134**	**85.35** ^**b**^	**7.84**		
	**Prehypertension**	**626**	**86.34** ^**b**^	**7.59**		
	**Hypertension**	**168**	**86.77** ^**b**^	**9.76**		
	**Total**	**3041**	**84.89**	**8.39**		
**Waist Circumference** ^**1)**^	**Normal**	**1112**	**0.50** ^**a**^	**0.05**	**21.348**	**< .001**
	**Elevated**	**1134**	**0.52** ^**b**^	**0.05**		
	**Prehypertension**	**626**	**0.52** ^**b**^	**0.05**		
	**Hypertension**	**168**	**0.52** ^**b**^	**0.06**		
	**Total**	**3040**	**0.51**	**0.05**		
**Body Fat Percentage (%)**	**Normal**	**2024**	**24.58** ^**a**^	**6.69**	**27.204**	**< .001**
	**Elevated**	**1910**	**25.62** ^**b**^	**6.17**		
	**Prehypertension**	**1019**	**26.46** ^**c**^	**6.06**		
	**Hypertension**	**300**	**26.94** ^**c**^	**6.11**		
	**Total**	**5253**	**25.46**	**6.40**		

Waist-to-height ratio

a<b<c<d: Tukey’s HSD

For females, the Prehypertension group (58.59±7.73) and Hypertension group (58.59±8.88) appeared to have greater body weight(p < .001). For body fat percentage, the Hypertension group recorded (36.11±6.68), and for BMI, the Prehypertension group recorded (25.10±3.06) while the Hypertension group recorded (25.25±3.63). In short, the Hypertension group was found out to have high figures in terms of body fat percentage(p < .001) and BMI(p < .001). In regards to obesity rate measured with waist circumference and waist-to-height ratio, the Prehypertension group and Hypertension group showed a high figure(p < .001). For waist circumference, the Prehypertension group recorded 83.82±7.76, while the Hypertension group recorded 83.60±9.52. For waist-to-height circumference, the two groups recorded 0.55±0.05 and 0.55±0.06, respectively (p < .001) (Table 3).

**Table 3:** Analysis of differences in physique by female blood pressure group.

		N	Mean	*SD*	*F*	*p*
**Height (cm)**	**Normal**	**4923**	**152.82**	**5.36**	**1.482**	**.217**
	**Elevated**	**4402**	**152.64**	**5.34**		
	**Prehypertension**	**1997**	**152.75**	**5.42**		
	**Hypertension**	**525**	**152.42**	**5.52**		
	**Total**	**11847**	**152.72**	**5.37**		
**Body Weight (kg)**	**Normal**	**4921**	**56.34** ^**a**^	**7.54**	**48.383**	**< .001**
	**Elevated**	**4403**	**57.38** ^**b**^	**7.80**		
	**Prehypertension**	**1998**	**58.59** ^**c**^	**7.73**		
	**Hypertension**	**524**	**58.59** ^**c**^	**8.88**		
	**Total**	**11846**	**57.20**	**7.78**		
**BMI (kg/m** ^**2**^ **)**	**Normal**	**4925**	**24.12** ^**a**^	**2.97**	**64.136**	**< .001**
	**Elevated**	**4404**	**24.62** ^**b**^	**3.03**		
	**Prehypertension**	**1998**	**25.10** ^**c**^	**3.06**		
	**Hypertension**	**525**	**25.25**_**c**_	**3.63**		
	**Total**	**11852**	**24.52**	**3.06**		
**Waist Circumference (cm)**	**Normal**	**2753**	**81.42** ^**a**^	**8.09**	**28.352**	**< .001**
	**Elevated**	**2414**	**82.47** ^**b**^	**7.98**		
	**Prehypertension**	**1228**	**83.82** ^**c**^	**7.76**		
	**Hypertension**	**309**	**83.60** ^**c**^	**9.52**		
	**Total**	**6704**	**82.34**	**8.11**		
**Waist Circumference** ^**1) **^	**Normal**	**2752**	**0.53** ^**a**^	**0.05**	**32.040**	**< .001**
	**Elevated**	**2413**	**0.54** ^**b**^	**0.05**		
	**Prehypertension**	**1228**	**0.55** ^**c**^	**0.05**		
	**Hypertension**	**309**	**0.55** ^**c**^	**0.06**		
	**Total**	**6702**	**0.54**	**0.05**		
**Body Fat Percentage (%)**	**Normal**	**4923**	**33.84a**	**7.70**	**35.814**	**< .001**
	**Elevated**	**4404**	**34.63b**	**6.49**		
	**Prehypertension**	**1997**	**35.41c**	**6.26**		
	**Hypertension**	**524**	**36.11d**	**6.68**		
	**Total**	**11848**	**34.50**	**7.02**		

Waist-to-height ratio

a<b<c<d: Tukey’s HSD

### 3.3 Analysis of differences in physical strength

#### 3.3.1 Health-related physical strength

Male seniors showed a statistically significant difference in muscular strength(p = .026) and flexibility(p < .001), and the Normal group showed a high figure (50.71±10.09) in muscular strength. For flexibility, the Normal group recorded low (3.55±10.01), but the Prehypertension group recorded high (5.45±9.32) (Table 4).

**Table 4:** Analysis of differences in health-related physical strength by male blood pressure group.

		N	Mean	*SD*	*F*	*p*
**Cardiorespiratory Endurance** ^**1)**^	**Normal**	**1912**	**107.29**	**25.25**	**0.902**	**.439**
	**Elevated**	**1825**	**108.17**	**25.46**		
	**Prehypertension**	**972**	**107.80**	**23.12**		
	**Hypertension**	**289**	**105.84**	**26.51**		
	**Total**	**4998**	**107.63**	**25.01**		
**Muscular Strength**	**Normal**	**2025**	**50.71**	**10.09**	**3.079**	**.026**
	**Elevated**	**1911**	**49.83**	**9.73**		
	**Prehypertension**	**1019**	**49.85**	**9.96**		
	**Hypertension**	**300**	**50.03**	**9.79**		
	**Total**	**5255**	**50.19**	**9.92**		
**Muscular Endurance** ^**2)**^	**Normal**	**2018**	**20.64**	**6.59**	**1.025**	**.380**
	**Elevated**	**1907**	**20.88**	**6.41**		
	**Prehypertension**	**1015**	**20.48**	**6.07**		
	**Hypertension**	**297**	**20.91**	**6.52**		
	**Total**	**5237**	**20.71**	**6.43**		
**Flexibility**	**Normal**	**2011**	**3.55*a***	**10.01**	**8.811**	**< .001**
	**Elevated**	**1907**	**3.96*a***	**9.94**		
	**Prehypertension**	**1016**	**5.45*b***	**9.32**		
	**Hypertension**	**298**	**4.56*ab***	**9.83**		
	**Total**	**5232**	**4.13**	**9.87**		

1) Two-minute Walk-in-place, 2) Sit-to-stand

a<b<c<d: Tukey’s HSD

Female seniors showed a statistically significant difference in cardiorespiratory endurance(p < .001), muscular strength(p = .025), and muscular endurance(p < .001). The Normal group recorded high cardiorespiratory endurance (103.87±24.62), followed with the elevated group (102±24.81). For muscular endurance, the Normal group showed a high record (18.90±5.99), while the figure was low in the Prehypertension group (18.20±6.20) (Table 5).

**Table 5:** Analysis of differences in health-related physical strength by female blood pressure group.

		N	Mean	*SD*	*F*	*p*
**Cardiorespiratory Endurance** ^**1)**^	**Normal**	**4661**	**103.87** ^**c**^	**24.62**	**14.505**	**< .001**
	**Elevated**	**4182**	**102.00** ^**bc**^	**24.81**		
	**Prehypertension**	**1893**	**99.92** ^**ab**^	**24.98**		
	**Hypertension**	**491**	**99.32** ^**a**^	**27.00**		
	**Total**	**11227**	**102.31**	**24.90**		
**Muscular Strength**	**Normal**	**4920**	**37.62**	**8.60**	**3.112**	**.025**
	**Elevated**	**4402**	**37.65**	**9.99**		
	**Prehypertension**	**1997**	**36.93**	**11.03**		
	**Hypertension**	**525**	**37.21**	**8.61**		
	**Total**	**11844**	**37.50**	**9.58**		
**Muscular Endurance** ^**2)**^	**Normal**	**4909**	**18.90** ^**b**^	**5.99**	**6.552**	**< .001**
	**Elevated**	**4384**	**18.70** ^**ab**^	**5.89**		
	**Prehypertension**	**1982**	**18.20** ^**a**^	**6.03**		
	**Hypertension**	**524**	**18.62** ^**ab**^	**5.83**		
	**Total**	**11799**	**18.70**	**5.96**		
**Flexibility**	**Normal**	**4912**	**13.75**	**8.03**	**0.298**	**.827**
	**Elevated**	**4390**	**13.63**	**7.89**		
	**Prehypertension**	**1994**	**13.66**	**7.67**		
	**Hypertension**	**524**	**13.50**	**8.56**		
	**Total**	**11820**	**13.68**	**7.94**		

1) Two-minute Walk-in-place, 2) Sit-to-stand

a<b<c<d: Tukey’s HSD

### 3.3.2 Skill-related physical strength

Male seniors did not show a statistically significant difference in skill-related physical strength by blood pressure levels (Table 6), but female seniors showed a statistically significant difference in motor coordination and balance. A high record of motor coordination was shown in the Hypertension group (26.88±6.53) and Elevated group (26.67±6.40), Prehypertension group (26.56±6.45), and normal group (25.91±6.45); the Hypertension group showed a high record in balance (6.72±1.82) (Table 7).

**Table 6:** Analysis of differences in skill-related physical strength by male blood pressure group.

		N	Mean	*SD*	*F*	*p*
**Motor Coordination1)**	**Normal**	**2006**	**25.19**	**6.33**	**0.473**	**.*701***
	**Elevated**	**1893**	**25.35**	**6.06**		
	**Prehypertension**	**1015**	**25.12**	**6.43**		
	**Hypertension**	**297**	**25.45**	**6.54**		
	**Total**	**5211**	**25.25**	**6.26**		
**Balance** ^**2)**^	**Normal**	**2015**	**6.11**	**1.56**	**0.831**	**.*477***
	**Elevated**	**1905**	**6.13**	**1.51**		
	**Prehypertension**	**1015**	**6.14**	**1.49**		
	**Hypertension**	**299**	**6.25**	**1.51**		
	**Total**	**5234**	**6.13**	**1.53**		

1) Figure-of-8 Walk, 2) Timed Up and Go

**Table 7:** Analysis of differences in skill-related physical strength by female blood pressure group.

		N	Mean	SD	F	p
**Motor Coordination1)**	**Normal**	**4889**	**25.91** ^**a**^	**6.45**	**13.122**	**< .001**
	**Elevated**	**4379**	**26.67** ^**b**^	**6.40**		
	**Prehypertension**	**1981**	**26.56** ^**b**^	**6.45**		
	**Hypertension**	**522**	**26.88** ^**b**^	**6.53**		
	**Total**	**11771**	**26.34**	**6.45**		
**Balance** ^**2)**^	**Normal**	**4912**	**6.45**_**a**_	**1.78**	**14.023**	**< .001**
	**Elevated**	**4392**	**6.56** ^**ab**^	**1.71**		
	**Prehypertension**	**1984**	**6.74** ^**bc**^	**2.12**		
	**Hypertension**	**525**	**6.72** ^**c**^	**1.82**		
	**Total**	**11813**	**6.55**	**1.82**		

1) Figure-of-8 Walk, 2) Timed Up and Go

a<b<c<d: Tukey’s HSD

## 4 Discussion

With With an aim to identify the relationship between physique and physical fitness by blood pressure levels of Korean seniors, this study utilized the physical fitness test results of the National Fitness Award 100 conducted in 2017, analyzing the data of 17,110 subjects aged 65 or above (11,855 females, 5,255 males).

Essential (primary) hypertension is mainly caused by living a problematic lifestyle, including lack of physical activity and a wrong diet [47, 48], which can accompany diseases such as obesity. Not all hypertensive patients are obese indeed, however, as there is a positive correlation between hypertension and obesity index [49], this study analyzed the relationship between physique and blood pressure.

As a result, both males and females showed a significant difference in body weight, body fat percentage, BMI, waist circumference, and waist-to-height ratio, while only males showed a significant difference in height. Such results are in line with the research that taller height leads to increased blood pressure for males more than females [50, 51]. Also, the obesity index (BMI, waist circumference, and waist-to-height ratio) increased as the groups of both gender had higher blood pressure, which is in line with the research result which reported that body mass index, waist-to-hip ratio, and waist circumference are higher in the prehypertension group than the normal group [52]. The findings of this study also suggest that obesity is a key predictor of hypertension.

Among the factors for improving life habits, the relationship with health conditions such as physical activity and physical strength, and blood pressure and diabetes have been proven to be highly correlated [53, 54]. As explained, since physical activity not only improves diseases but also improves physical strength, this study investigated various health-related physical strength factors according to blood pressure level. As a result, females showed a significant difference in cardiorespiratory endurance, muscular strength, and muscular endurance, while males appeared to have a significant difference in muscular strength and flexibility. Such results were in line with the research that found out systolic pressure, diastolic pressure, average arterial pressure, and cardiorespiratory endurance are in a significant negative correlation [55], and the study that the systolic pressure of aged females between 65–74 is in a negative correlation with cardiorespiratory endurance [56]. This implies that even though high cardiorespiratory endurance cannot entirely prevent the development of hypertension, higher cardiorespiratory endurance can lower the risk of hypertension [57].

A decrease in mean arterial blood pressure during the recovery period after maximum intensity exercise is greater in women, and it was suggested that the decrease in arterial blood pressure of women was due to a greater vasoconstrictor response [58]. Therefore, it was reported that in women, total peripheral resistance is increased by compensatory vasoconstrictor action for a decrease in cardiac output and mean arterial blood pressure [59], and it was identified that strategies for controlling blood pressure of women through exercise are considered to be different from men because the difference in cardiovascular responses to control blood pressure during incremental intensity exercise differ according to gender [60].

Preceding studies have highlighted muscular strength as a physical strength factor to reduce blood pressure as the weakened grip strength among the oldest old leads to increased blood pressure [56]. Also, other preceding research results on the correlation of muscular strength and blood pressure stated that muscular strength was shown to be significantly low in the Prehypertension and Hypertension groups when compared with the Normal group in all age groups [61], and one study on people aged 50–75 reported that systolic pressure has a negative correlation with muscular strength [62]. The findings of this study found out that both male and female subjects showed a significant difference in muscular strength by blood pressure levels, but it was just that the Normal group showed a high level of muscular strength, and no clear pattern based on blood pressure levels was shown. Also, for muscular endurance, females in the Normal group appeared to have the best muscular endurance, showing a trend where the higher blood pressure level led to a significant drop in muscular endurance. Such observations are in line with the study that reported muscular endurance decreases with higher blood pressure [49], but as males did not show a significant difference, it is considered that there needs to be further studies conducted on muscular strength.

The ACSM recommends hypertensive patients to carry out flexibility exercises 2 to 3 times a week along with aerobic and resistance exercises in its guidelines [63]. Also, it is reported that high flexibility can lower the prevalence of hypertension [64]. In regards to flexibility, only males showed a significant difference by blood pressure, where the Prehypertension group appeared to have the greatest flexibility and the Normal group had the lowest. Such results were identical to the research that flexibility increased significantly in the order of Prehypertension group, Elevated group, and Normal group for males aged 60 or older [61]. Flexibility is an important physical fitness factor for muscle stretch and joint mobilization [65], but for aged males, blood pressure and flexibility were in a negative relationship.

Age-related increase in systolic blood pressure and pulse pressure accelerates after middle age [66]. Similarly, trunk flexibility has been reported to deteriorate faster after middle age, based on 6000 flexibility results [67]. Blood pressure is dependent on arterial wall stiffness, and collagen fibers in the arterial walls are involved in the stiffness [68]. In addition, when comparing the physical fitness of men and women of the same age, flexibility is the only measure of physical fitness where women score higher than men [69]. Women also generally have lower blood pressure than men of the same age [66]. These factors may be related to the fact that women have less connective tissue collagen [70] and a much less age-related effect on the stiffness of collagen material parameters in women [71]. Clarifying these mechanisms requires further research.

As in this study, it is not easy to compare preliminary studies with this study because the research on various physical factors according to blood pressure level is very insufficient. However, in a similar preliminary study [49], muscular strength, muscular endurance, balance, flexibility, and cardiovascular endurance of normal blood pressure group, early high blood pressure group, and stage 1 high blood pressure group were compared, and the results of the study identified that early high blood pressure group has the best muscular strength and flexibility. Also, in the study by So & Choi [43], the difference in physical strength level according to high blood pressure standards was investigated, and as a result, men in their 60s, early high blood pressure group showed the best results in muscular endurance, speed, agility, and balance. In a study by Kim et al. [72] conducted in 2022 on men and women over 65 years of age(218,848 people) that measured muscular strength, muscular endurance, cardiovascular endurance, balance, coordination, and flexibility and gave Level 1.

In a study conducted by Kim, Bong Ju et al in 2022 on men and women over 65 years of age(218,848 people) that measured muscular strength, muscular endurance, cardiovascular endurance, balance, coordination, and flexibility and gave level 1 if above 70th quintile, level 2 for above 50th quintile, level 3 for above 30th quintile, and the rest level 4, the result turned out to be the opposite of what is generally expected that the blood pressure would be lower when fitness level is hgher. It is believed that follow-up studies are needed for clearer identification because there are still various opinions in the studies on flexibility.

According to the results of analyzing skill-related physical fitness by blood pressure, males did not show differences between groups, while females showed differences in motor coordination and balance. Females’ motor coordination and balance were all higher in the Elevated, Prehypertension, and Hypertension groups than the Normal group. In other words, females with higher blood pressure can be regarded to have poor motor coordination and balance. For balance, those aged between 65–75 showed a significant positive correlation between systolic pressure and balance [56], which was in line with the research which reported that balance (p = 0.006) increased with higher blood pressure [49]. Furthermore, among the preceding research on motor coordination and balance after exercise intervention conducted on hypertensive patients, studies that showed that the systolic pressure of the Hypertension group significantly dropped after participating in an exercise program and that motor coordination and balance significantly increased making improvements [73] support the findings of this study.

In sum, males only showed a significant difference in muscular strength and flexibility, while aged females showed a significant difference between groups in all categories except flexibility. It is necessary to develop various gender-based exercise programs that take other physical factors of women and men into account because women have multiple body functions that decrease faster than men [49, 62–64] and have a higher rate of dysfunction than men [74]. In particular, it is believed that exercise prescription for controlling blood pressure of women should be made differently from men because women show a higher heart rate change than men to maintain cardiac output that serves to supply oxygen and nutrients to the muscles that are active during exercise.

Lastly, the 8th report of the Joint National Committee, which suggests guidelines for the treatment and management of hypertension, recommends patients to consistently carry out lifestyle interventions across their overall efforts for managing health. It is important to create a healthy lifestyle for the management of hypertension [5]. In particular, as regular exercise is told to be a safer way of lowering blood pressure compared to drug treatment [75], it is considered that various exercise programs need to be developed and suggested to hypertensive patients, on top of aerobic exercise, reflecting physical strength factors and individual preferences as proved in this study to more effectively prevent and improve hypertension.

## 5 Conclusion

This study intended to investigate the association with various physical strength factors that can help to improve blood pressure of the elderly by analyzing health, technology-related physical strength, and physique by blood pressure level of the elderly in Korea, As a result of the study, it was identified that the blood pressure level of the elderly differed in physical strength factors according to gender.

As the results of the study, the blood pressure levels of seniors differed greatly by physical strength factors by gender. While males showed a significant difference in only muscular strength and flexibility, females showed a significant difference between groups in health and skill-related and physical fitness (cardiorespiratory endurance, muscular strength, balance, motor coordination) except flexibility, which implied that they are valid indicators for the management of blood pressure. Hence, the results of the study are expected to be used as primary data for the development of exercise programs for the prevention and improvement of hypertension considering the gender difference and various physical strength factors. However, there were limitations as the study was cross-sectional research, and comorbidities and treatment history could not be identified. There is a need for an epidemiological study to examine the association.

## References

[1] Whaley-Connell A., McCullough P. A., & Sowers J. R. (2011). The role of oxidative stress in the metabolic syndrome. Reviews in cardiovascular medicine, 12(1), 21–29. doi: 10.3909/ricm0555 21546885

[2] World Health Organization. Hypertension. 2019. https://www.who.int/news-room/fact-sheets/detail/hypertension (25 August 2021)

[3] Ingaramo R. A. (2016). Obesity, diabetes, and other cardiovascular risk factors in native populations of South America. Current hypertension reports, 18(1), 1–5. doi: 10.1007/s11906-015-0613-6 26780771

[4] Mertens W. C., Eisenhauer E. A., Jolivet J., Ernst S., Moore M., & Muldal A. (1994). Docetaxel in advanced renal carcinoma: A phase II trial of the National Cancer Institute of Canada Clinical Trials Group. Annals of oncology, 5(2), 185–187. 10.1093/oxfordjournals.annonc.a058776 7910480

[5] James P. A., Oparil S., Carter B. L., Cushman W. C., Dennison-Himmelfarb C., Handler J., et al. (2014). 2014 evidence-based guideline for the management of high blood pressure in adults: report from the panel members appointed to the Eighth Joint National Committee (JNC 8). Jama, 311(5), 507–520. doi: 10.1001/jama.2013.284427 24352797

[6] Diaz K. M., & Shimbo D. (2013). Physical activity and the prevention of hypertension. Current hypertension reports, 15(6), 659–668. doi: 10.1007/s11906-013-0386-8 24052212 PMC3901083

[7] Cheng H. M., Chiang C. E., & Chen C. H. (2015). The novelty of the 2015 guidelines of the Taiwan society of cardiology and the Taiwan hypertension society for the management of hypertension. Pulse, 3(1), 29–34. doi: 10.1159/000381299 26587455 PMC4646150

[8] Statistics Korea: High Blood Pressure Prevalence Trend. 2021. https://kdca.go.kr. (13. May. 2021)

[9] Poulter NR, Prabhakaran D, Caulfield M. Hypertension. Lancet. 2015; 386(9995). pp. 801–812. doi: 10.1016/S0140-6736(14)61468-9 25832858

[10] Kim O. S., Jeon H. O., Kim D. H., Kim B. H., & Kim H. J. (2009). Risk factors of prehypertension in Korean adults: The Korean National Health and Nutrition Examination Survey 2005. Korean Journal of Adult Nursing, 21(3), 281–292.

[11] Svetkey L. P., Harsha D. W., Vollmer W. M., Stevens V. J., Obarzanek E., Elmer P. J., et al. (2003). Premier: a clinical trial of comprehensive lifestyle modification for blood pressure control: rationale, design and baseline characteristics. Annals of epidemiology, 13(6), 462–471. doi: 10.1016/s1047-2797(03)00006-1 12875806

[12] Kim J. Y., Wineinger N. E., & Steinhubl S. R. (2016). The influence of wireless self-monitoring program on the relationship between patient activation and health behaviors, medication adherence, and blood pressure levels in hypertensive patients: a substudy of a randomized controlled trial. Journal of medical Internet research, 18(6), e5429. doi: 10.2196/jmir.5429 27334418 PMC4935792

[13] Brook R. D., & Rajagopalan S. (2018). 2017 ACC/AHA/AAPA/ABC/ACPM/AGS/APhA/ASH/ASPC/NMA/PCNA Guideline for the Prevention, Detection, Evaluation, and Management of High Blood Pressure in Adults. A report of the American College of Cardiology/American Heart Association Task Force on Clinical Practice Guidelines. Journal of the American Society of Hypertension: JASH, 12(3), 238–238. doi: 10.1016/j.jash.2018.01.004 29396104

[14] Korea Hypertension Management Association. High blood pressure health common sense [internet]. Available: http://www.khma.or.kr/site/?doc_sub=02_06.

[15] Ben-Sira D., & Oliveira J. M. F. (2007). Hypertension in aging: physical activity as primary prevention. European Review of Aging and Physical Activity, 4(2), 85–89. doi: 10.1007/s11556-007-0023-0

[16] Svetkey L. P., Pollak K. I., Yancy W. S. Jr, Dolor R. J., Batch B. C., Samsa G., et al. (2009). Hypertension improvement project: randomized trial of quality improvement for physicians and lifestyle modification for patients. Hypertension, 54(6), 1226–1233. doi: 10.1161/HYPERTENSIONAHA.109.134874 19920081 PMC2784648

[17] BAZARGAN H. S., ANI C., Gaines T., AHMADI A. R., & Bazargan M. (2010). Alcohol misuse and depression symptoms among males and females. 20597567

[18] Ministry of Health and Welfare (2005). Korea National Health and Nutrition Examination Survey.

[19] Suri M. F. & Qureshi A. I.(2006). Prehypertension as a Risk Factor for Cardiovascular Disease. J Cardiovasc Nurs. 21(6), 478–82 17293739 10.1097/00005082-200611000-00012

[20] Maruthur N. M., Wang N. Y., & Appel L. J. (2009). Lifestyle interventions reduce coronary heart disease risk: results from the PREMIER Trial. Circulation, 119(15), 2026–2031. doi: 10.1161/CIRCULATIONAHA.108.809491 19349322 PMC2995494

[21] Yoon P. W., Bastian B., Anderson R. N., Collins J. L., & Jaffe H. W. (2014). Potentially preventable deaths from the five leading causes of death—United States, 2008–2010. Morbidity and Mortality Weekly Report, 63(17), 369 24785982 PMC4584887

[22] Chobanian A. V.·Bakris G. L. & Black H. R., Cus(2003). The seventh report of the joint national committee on prevention, detection, evaluation, and treatment of high blood pressure. JAMA, 289(19), 1206–1256. 10.1001/jama.289.19.2560 12748199

[23] Bardage C., & Isacson D. G. (2001). Hypertension and health-related quality of life: an epidemiological study in Sweden. Journal of clinical epidemiology, 54(2), 172–181. 10.1016/S0895-4356(00)00293-6 11166533

[24] Jennings G. L. (2010). Hypertension and adherence to physical activity programs—a sticky matter!. British journal of sports medicine, 44(14), 994–997. doi: 10.1136/bjsm.2009.067306 19846419

[25] Börjesson M., Onerup A., Lundqvist S., & Dahlöf B. (2016). Physical activity and exercise lower blood pressure in individuals with hypertension: narrative review of 27 RCTs. British journal of sports medicine, 50(6), 356–361. doi: 10.1136/bjsports-2015-095786 26787705

[26] Swift D. L., McGee J. E., Earnest C. P., Carlisle E., Nygard M., & Johannsen N. M. (2018). The effects of exercise and physical activity on weight loss and maintenance. Progress in cardiovascular diseases, 61(2), 206–213. doi: 10.1016/j.pcad.2018.07.014 30003901

[27] Choi S. W. (2005). Effects of aerobic and resistance training on the health related physical fitness and blood components in elderly women. Korean Journal of Sports Science, 14(2), pp. 679–691.

[28] Blair S. N., Kohl H. W., Paffenbarger R. S., Clark D. G., Cooper K. H., & Gibbons L. W. (1989). Physical fitness and all-cause mortality: a prospective study of healthy men and women. Jama, 262(17), 2395–2401. doi: 10.1001/jama.1989.03430170057028 2795824

[29] Meyers J. (2002). Prakash M, Froelicher V, Do D, Partington S, Atwood J. Exercise capacity and mortality amount men referred for exercise testing. N Engl J Med, 346, 793–801. doi: 10.1056/NEJMoa011858 11893790

[30] Mora S., Redberg R. F., Cui Y., Whiteman M. K., Flaws J. A., Sharrett A. R., et al. (2003). Ability of exercise testing to predict cardiovascular and all-cause death in asymptomatic women: a 20-year follow-up of the lipid research clinics prevalence study. Jama, 290(12), 1600–1607. doi: 10.1001/jama.290.12.1600 14506119

[31] Metter E. J., Talbot L. A., Schrager M., & Conwit R. (2002). Skeletal muscle strength as a predictor of all-cause mortality in healthy men. The Journals of Gerontology Series A: Biological Sciences and Medical Sciences, 57(10), B359–B365. 10.1093/gerona/57.10.B359 12242311

[32] Beckett N. S., Peters R., Fletcher A. E., Staessen J. A., Liu L., Dumitrascu D., et al. (2008). Treatment of hypertension in patients 80 years of age or older. New England Journal of Medicine, 358(18), 1887–1898. doi: 10.1056/NEJMoa0801369 18378519

[33] Pescatello L. S., MacDonald H. V., Ash G. I., Lamberti L. M., Farquhar W. B., Arena R., et al. (2015, June). Assessing the existing professional exercise recommendations for hypertension: a review and recommendations for future research priorities. In Mayo Clinic Proceedings (Vol. 90, No. 6, pp. 801–812). Elsevier. 10.1016/j.mayocp.2015.04.008 26046413

[34] Forouzanfar M. H., Afshin A., Alexander L. T., Anderson H. R., Bhutta Z. A., Biryukov S., et al. (2016). Global, regional, and national comparative risk assessment of 79 behavioural, environmental and occupational, and metabolic risks or clusters of risks, 1990–2015: a systematic analysis for the Global Burden of Disease Study 2015. The lancet, 388(10053), 1659–1724. doi: 10.1016/S0140-6736(16)31679-8 27733284 PMC5388856

[35] Baek S. H. Choi, S. W. (2012). Effect of 12-weeks regular exercise program on improving, blood pressure & b-aPWV of adult with high risks of atherosclerosis. Korean Journal of Sports Science, 21(3), 1017–1025.

[36] Molmen-Hansen H. E., Stolen T., Tjonna A. E., Aamot I. L., Ekeberg I. S., Tyldum G. A., et al. (2012). Aerobic interval training reduces blood pressure and improves myocardial function in hypertensive patients. European journal of preventive cardiology, 19(2), 151–160. doi: 10.1177/1741826711400512 21450580

[37] Dimeo F., Pagonas N., Seibert F., Arndt R., Zidek W., & Westhoff T. H. (2012). Aerobic exercise reduces blood pressure in resistant hypertension. Hypertension, 60(3), 653–658. doi: 10.1161/HYPERTENSIONAHA.112.197780 22802220

[38] Bakker E. A., Sui X., Brellenthin A. G., & Lee D. C. (2018). Physical activity and fitness for the prevention of hypertension. Current Opinion in Cardiology, 33(4), 394–401. doi: 10.1097/HCO.0000000000000526 29762150

[39] Carnethon M. R., Gidding S. S., Nehgme R., Sidney S., Jacobs D. R. Jr, & Liu K. (2003). Cardiorespiratory fitness in young adulthood and the development of cardiovascular disease risk factors. Jama, 290(23), 3092–3100. doi: 10.1001/jama.290.23.3092 14679272

[40] Katzmarzyk P. T., Church T. S., & Blair S. N. (2004). Cardiorespiratory fitness attenuates the effects of the metabolic syndrome on all-cause and cardiovascular disease mortality in men. Archives of internal medicine, 164(10), 1092–1097. doi: 10.1001/archinte.164.10.1092 15159266

[41] Stevens J., Cai J., Evenson K. R., & Thomas R. (2002). Fitness and fatness as predictors of mortality from all causes and from cardiovascular disease in men and women in the lipid research clinics study. American journal of epidemiology, 156(9), 832–841. doi: 10.1093/aje/kwf114 12397001

[42] Wei M. Y., & Mukamal K. J. (2018). Multimorbidity, mortality, and long-term physical functioning in 3 prospective cohorts of community-dwelling adults. American journal of epidemiology, 187(1), 103–112. doi: 10.1093/aje/kwx198 29309518 PMC5860284

[43] Ezzatvar Y., Ramírez-Vélez R., Sáez de Asteasu M. L., Martínez-Velilla N., Zambom-Ferraresi F., Izquierdo M., et al. (2021). Physical function and all-cause mortality in older adults diagnosed with cancer: a systematic review and meta-analysis. The Journals of Gerontology: Series A, 76(8), 1447–1453. 10.1093/gerona/glaa305 33421059

[44] Kim H. J., & Park J. S. (2020). The effects of Aerobic exercise intensity participation training on body composition, health related fitness and quality of life in elderly women. Korean Journal of Sport Science, 31(1), 35–47. 10.24985/kjss.2020.31.1.35

[45] The Korean Society of Hypertension. 2018 Medical guidelines for high blood pressure.

[46] Korea Institute of Sport Science. 2012. Development of national physical strength certification standards for the elderly

[47] Gurven M., Blackwell A. D., Rodríguez D. E., Stieglitz J., & Kaplan H. (2012). Does blood pressure inevitably rise with age? Longitudinal evidence among forager-horticulturalists. Hypertension, 60(1), 25–33. doi: 10.1161/HYPERTENSIONAHA.111.189100 22700319 PMC3392307

[48] Kokkinos P. (2014). Cardiorespiratory fitness, exercise, and blood pressure. Hypertension, 64(6), 1160–1164. doi: 10.1161/HYPERTENSIONAHA.114.03616 25245388

[49] Kang S. H., & So W. Y. (2018). Difference in physical fitness level depending on hypertension and body mass index in the elderly. Korean J Adapt Phys Act, 26, 85–94.

[50] Rosner B., Cook N. R., Daniels S., & Falkner B. (2013). Childhood blood pressure trends and risk factors for high blood pressure: the NHANES experience 1988–2008. Hypertension, 62(2), 247–254. doi: 10.1161/HYPERTENSIONAHA.111.00831 23856492 PMC3769135

[51] Dong B., Wang Z., Wang H. J., & Ma J. (2015). Blood pressure-to-height ratio for screening prehypertension and hypertension in Chinese children. Journal of Human Hypertension, 29(10), 618–622. doi: 10.1038/jhh.2014.133 25631223

[52] Tsai P. S., Ke T. L., Huang C. J., Tsai J. C., Chen P. L., Wang S. Y., et al. (2005). Prevalence and determinants of prehypertension status in the Taiwanese general population. Journal of hypertension, 23(7), 1355–1360. doi: 10.1097/01.hjh.0000173517.68234.c3 15942457

[53] Francesconi C., Lackinger C., Weitgasser R., Haber P., & Niebauer J. (2016). Physical activity and exercise training in the prevention and therapy of type 2 diabetes mellitus. Wiener Klinische Wochenschrift, 128, S141–5. doi: 10.1007/s00508-015-0923-3 27052239

[54] Lou M., Zong X. F., & Wang L. L. (2017). Curative treatment of hypertension by physical exercise. Eur Rev Med Pharmacol Sci, 21(14), 3320–6. 28770948

[55] Parkhad S. B., & Palve S. B. (2014). Association of physical activity and physical fitness with blood pressure profile in Maharashtrian adolescent boys and girls. Internet Journal of Medical Update, 9(1), 4–9. https://link.gale.com/apps/doc/A365458613/HRCA?u=anon~7ca8d936&sid=googleScholar&xid=af552ce9

[56] Jung S. M., Cho W. J. (2018) The Effect of Body Composition and Functional Fitness on Prevalence of Hypertension according to Gender and the Elderly. Korean Journal of Sports Science, 14(2), 679–691. doi: 10.35159/kjss.2018.12.27.6.1275

[57] Crump C., Sundquist J., Winkleby M. A., & Sundquist K. (2016). Interactive effects of physical fitness and body mass index on the risk of hypertension. JAMA internal medicine, 176(2), 210–216. doi: 10.1001/jamainternmed.2015.7444 26784837 PMC4803286

[58] Carter R. 3rd., Wilson T., Crandall C. G., Watenpaugh D. E., & Smith M. L. (2001). Effect of cutaneous vasodilation during dynamic exercise on arterial pressure regulation during inactive exercise recovery (Abstract). The Journal of the Federation of American Societies for Experimental Biology, 15: A789.

[59] Fadel B. M., Abdulbaki K., Nambiar V., Al Amri M., Shahid M., Khouqeer F., et al. (2007). Dual thrombosis of the pulmonary arterial and venous anastomotic sites after single lung transplantation: role of transesophageal echocardiography in diagnosis and management. Journal of the American Society of Echocardiography, 20(4), 438–e9. doi: 10.1016/j.echo.2006.10.024 17400127

[60] Arbab-Zadeh A., Dijk E., Prasad A., Fu Q., Torres P., Zhang R., et al. (2004). Effect of aging and physical activity on left ventricular compliance. Circulation, 110(13), 1799–1805. doi: 10.1161/01.CIR.0000142863.71285.74 15364801

[61] So W. Y., & Choi D. H. (2009). The difference of fitness level according to blood pressure in korean men. Korean Journal of Health Promotion and Disease Prevention, 9(2), 122–128.

[62] Gubelmann C., Vollenweider P., & Marques-Vidal P. (2017). Association of grip strength with cardiovascular risk markers. European journal of preventive cardiology, 24(5), 514–521. doi: 10.1177/2047487316680695 27885059

[63] Moore G., Durstine J. L., Painter P., & American College of Sports Medicine. (2016). Acsm’s exercise management for persons with chronic diseases and disabilities, 4E. Human Kinetics. doi: 10.3305/nh.2015.32.2.9228

[64] Gando Y., Sawada S. S., Momma H., Kawakami R., Miyachi M., Lee I. M., et al. (2021). Body flexibility and incident hypertension: The Niigata wellness study. Scandinavian Journal of Medicine & Science in Sports, 31(3), 702–709. doi: 10.1111/sms.13867 33141990

[65] Miller W. F., Johnson R. L. Jr, & Wu N. (1959). Relationships between fast vital capacity and various timed expiratory capacities. Journal of Applied Physiology, 14(2), 157–163. doi: 10.1152/jappl.1959.14.2.157 13641134

[66] Franklin S. S., Gustin IV W., Wong N. D., Larson M. G., Weber M. A., Kannel W. B., et al. (1997). Hemodynamic patterns of age-related changes in blood pressure: the Framingham Heart Study. Circulation, 96(1), 308–315. 10.1161/01.CIR.96.1.308 9236450

[67] Medeiros H. B. D. O., Araújo D. S. M. S. D., & Araújo C. G. S. D. (2013). Age-related mobility loss is joint-specific: an analysis from 6,000 Flexitest results. Age, 35(6), 2399–2407. doi: 10.1007/s11357-013-9525-z 23529505 PMC3824991

[68] Vlachopoulos C., O’Rourke M., & Nichols W. W. (2011). McDonald’s blood flow in arteries: theoretical, experimental and clinical principles. CRC press.

[69] Allander E., Björnsson O. J., Olafsson O., Sigfusson N., & Thorsteinsson J. (1974). Normal range of joint movements in shoulder, hip, wrist and thumb with special reference to side: a comparison between two populations. International journal of epidemiology, 3(3), 253–261. doi: 10.1093/ije/3.3.253 4414846

[70] Alter M. J. (2004). Science of flexibility. Human Kinetics.

[71] Åstrand H., Stalhand J., Karlsson J., Karlsson M., Sonesson B., & Länne T. (2011). In vivo estimation of the contribution of elastin and collagen to the mechanical properties in the human abdominal aorta: effect of age and sex. Journal of applied physiology, 110(1), 176–187. doi: 10.1152/japplphysiol.00579.2010 21071586

[72] Sung B. J., Seo J. W., Lee J. Y. (2022). Differences in Blood Pressure and Obesity According to the Physical Fitness Level for Korean Older Persons: Considering Data from the National Fitness 100. Korean Journal of Sport Science, 33(2), 161–168. 10.24985/kjss.2022.33.2.161

[73] Ko S. H., Park C. H., & Jekal Y. S. (2017). Effects of exercise intervention program on the level of obesity, physical fitness level and metabolic related risk factors among hypertensive and diabetic patients. Journal of the Korean society for Wellness, 12(1), 645–655. doi: 10.21097/ksw

[74] Puggaard L. (2003). Effects of training on functional performance in 65, 75 and 85 year‐old women: Experiences deriving from community based studies in Odense, Denmark. Scandinavian journal of medicine & science in sports, 13(1), 70–76. doi: 10.1034/j.1600-0838.2003.00302.x 12535320

[75] Loiola Souto A., Moreira Lima L., Castro E. A. D., Peixoto Veras R., Segheto W., Camargos Zanatta T., et al. (2015). Blood pressure in hypertensive women after aerobics and hydrogymnastics sessions. Nutrición Hospitalaria, 32(2), 823–828. doi: 10.3305/nh.2015.32.2.9228 26268117

